# The effect of resizing on the natural appearance of scintigraphic images: an image similarity analysis

**DOI:** 10.3389/fnume.2024.1505377

**Published:** 2025-02-06

**Authors:** Siraj Ghassel, Amir Jabbarpour, Jochen Lang, Eric Moulton, Ran Klein

**Affiliations:** ^1^ Electrical Engineering and Computer Science, University of Ottawa, Ottawa, ON, Canada; ^2^ Department of Physics, Carleton University, Ottawa, ON, Canada; ^3^ Jubilant DraxImage Inc., Kirkland, QC, Canada; ^4^ Division of Nuclear Medicine and Molecular Imaging, Faculty of Medicine, University of Ottawa, Ottawa, ON, Canada; ^5^ Department of Nuclear Medicine and Molecular Imaging, The Ottawa Hospital, Ottawa, ON, Canada

**Keywords:** nuclear medicine, scintigraphy, image processing, image resizing, image noise

## Abstract

**Background and objective:**

This study aimed to assess the impact of upsampling and downsampling techniques on the noise characteristics and similarity metrics of scintigraphic images in nuclear medical imaging.

**Methods:**

A physical phantom study using dynamic imaging was used to generate reproducible static images of varying count statistics. Naïve upsampling and downsampling with linear interpolation were compared against alternative methods based on the preservation of Poisson count statistics and principles of nuclear scintigraphic imaging; namely, linear interpolation with a Poisson resampling correction (upsampling) and a sliding window summation method (downsampling). For each resizing method, we computed the similarity of resized images to count-matched images acquired at the target grid size with the structural similarity index measure and the logarithm of the mean squared error. These image quality metrics were subsequently compared to those of two independent count-matched images at the target grid size (representing variance due to natural noise permutations) as a reference to establish an optimal resizing method.

**Results:**

Only upsampled images with the Poisson resampling correction after linear interpolation produced images that were similar to those acquired at the target grid size. For downsampling, both linear interpolation and sliding window summation yielded similar outcomes for a reduction factor of 2. However, for a reduction factor of 4, only sliding window summation resulted in image similarity metrics in agreement with those at the target grid size.

**Conclusions:**

The study underlines the importance of applying appropriate resizing techniques in nuclear medical imaging to produce realistic images at the target grid size.

## Introduction

Image resizing is a common image processing operation to resample an image from one grid size to another (1). When the image is upsampled (the pixel density increases), a choice of interpolation methods may be applied, the most common of which are nearest neighbor, bilinear, bicubic, and b-spline interpolation. When the image is downsampled (the pixel density decreases), the standard procedure recommends applying a low pass filter to prevent aliasing (2). While each interpolation method tries to maximize the similarity of the destination to the source image (3), they differ in how the pixel values in the neighborhood of the source coordinate are combined to calculate the final value at the destination coordinate.

In almost all imaging modalities, the process of resizing may not substantially alter the semantic nature of the image (4). However, in the case of nuclear medicine scintigraphy, where the native image unit is the number of detected events (i.e., photon counts) (5), Poisson counting statistics play a visually perceivable and mathematically significant role in the image noise (6). As dictated by Poisson counting statistics, the variance in the signal is equal to the mean (expected true counts) of the sample; hence, the relative noise decreases as the square root of the mean counts as shown in Equation 1.

(1) $$\mathrm{relative}\;\mathrm{noise}=\;\frac{\mathrm{standard}\;\mathrm{deviation}}{\mathrm{mean}}=\frac{\sqrt{\mathrm{mean}}}{\mathrm{mean}}=\frac{1}{\sqrt{\mathrm{mean}}}$$

Total photon counts, and thus photon density, need to be conserved if downstream operations are dependent on accurate photon counts or noise modeling. The advantages of proper image resizing include the preservation of critical image features such as photon counts and noise characteristics, which are vital for accurate diagnoses and clinical decision-making. Since resizing intrinsically modifies the number of pixels and pixel spacing (7), the resulting resized image should reflect the splitting or joining of counts from the original image in the target image when upsampling or downsampling, respectively. An accurate accounting of counts models what the image would have looked like had the image been acquired at the target spatial grid and corresponding pixel spacing, including the magnitude of the noise in each pixel (8).

**Figure 1:**
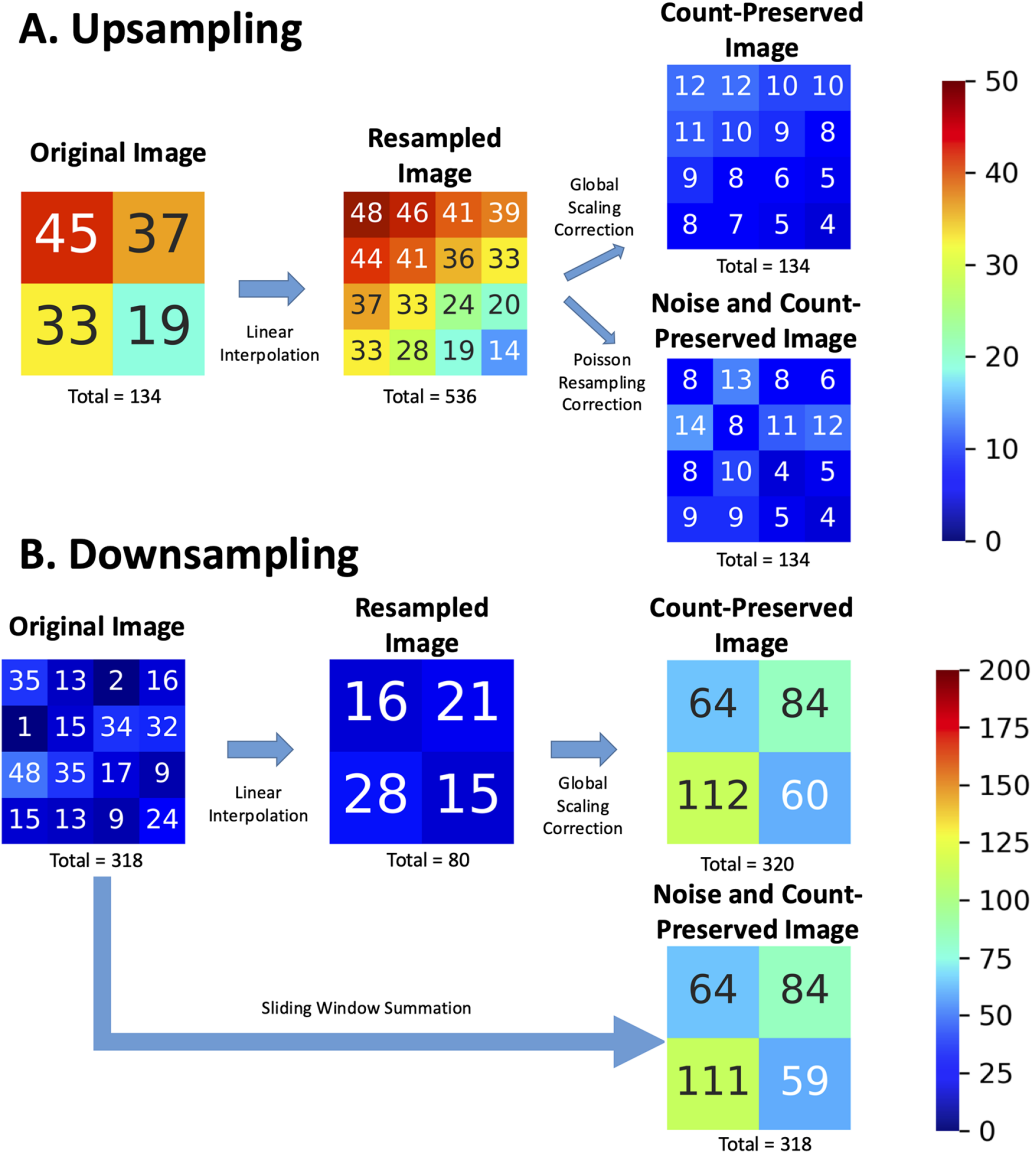
Example of **(A)** upsampling and **(B)** downsampling operations on nuclear scintigraphy images (original) with linear interpolation, showing the resulting images and incorrect total events. “Count-preserved images” obtained with a global scaling correction are contrasted with ideal “Noise and Count-preserved images” as if they had been acquired by an imaging system with the target pixel sizes and local noise associated with the increased or decreased counts per pixel. Numbers represent pixel intensity as event counts in the corresponding pixel and are rounded to the nearest integer.

To demonstrate upsampling (Figure 1A), a 2 × 2 grid representing pixel counts is resized to a 4 × 4 grid using linear interpolation. Each new pixel value in the 4 × 4 grid is computed as a weighted average of its neighboring pixels in the original 2 × 2 grid. This process increases the total number of pixels, but without further correction, it also inflates the total event counts by a factor of 2^2^ = 4. For instance, if the original 2 × 2 grid has a total count of 134, the uncorrected 4 × 4 grid may erroneously display a total count of 134 × 4 = 536 due to the increased pixel density. To address this, a global scaling correction is applied to the interpolated image, reducing the pixel values by a factor equal to the ratio of the old pixel area to the new pixel area [in this case, (2/4)^2^ = 1/4]. This ensures that the total counts remain consistent with the original image. In addition, a Poisson resampling correction may be applied to reduce the excess photon counts while emulating the natural Poisson noise associated with the lower counts in each pixel. In fact, White and Lawson (9) have demonstrated that Poisson resampling is the appropriate technique for artificially reducing counts in scintigraphic images. While this technique was originally intended to generate synthetic low-count scintigraphic images from high-count ones, this method can be reasonably repurposed to correct the added collateral counts from upsampling.

Downsampling, however, is analogous to acquiring an image on a smaller spatial grid with a larger pixel size. Properly implemented, this operation effectively corresponds to the summation of photon counts within a sliding window whose size is given by the downsampling ratio. This method will necessarily conserve the count statistics and Poisson noise of the resulting image, mimicking how a gamma camera would have aggregated photon counts within a larger pixel size. In Figure 1B, a 4 × 4 grid is resized to a 2 × 2 grid. Linear interpolation calculates each new pixel value in the 2 × 2 grid as the average of a 2 × 2 window from the original 4 × 4 grid. For instance, the original 4 × 4 image contains 318 counts, but the resampled image obtained through linear interpolation reduces this to approximately 80 counts—approximately four times fewer. To address this, a global scaling correction is applied [in this case (4/2)^2^ = 4], to restore the total event counts. In more concrete terms, if we use the example of a 256 × 256 image to be resized to 128 × 128, the resulting image should be the same as if the image had been natively acquired on a 128 × 128 imaging grid (within the acceptable limits of random noise associated with two independent image samples). Each resulting pixel is thus expected to have four times more counts (the sum of four pixels sampling the same corresponding image space) on average than the original image pixel. By directly summing pixels with an appropriately sized sliding window, rather than interpolating (averaging) and then factoring, we can accommodate downsampling by factors greater than two and avoid precision losses associated with integer rounding during interpolation.

Joint count and noise preservation are paramount for many image processing investigations in nuclear medicine (10, 11). Currently, considering the immense work in artificial intelligence (AI) model development in medical imaging (12, 13), AI developers may circumvent the high variability in image sizes in real-life clinical settings by forcing a model's input images to a fixed spatial grid under the assumption that these resized images reflect natively acquired ones on the destination spatial grid. This does not pose a problem for modalities such as magnetic resonance (MR) or computed tomography (CT), where the content of the image does not change after resizing; however, such is not the case in nuclear medicine where trivial resizing operations introduce false pixel count and/or noise representations in images.

Given the above, we used image data from a physical phantom to conduct three experiments. First, we generated synthetic low-count images from higher-count images to simulate varying levels of count statistics and validated this methodology against the phantom data. Second, we performed upsampling from low to high grid sized images using naïve linear interpolation (1) and Poisson resampling corrections and compared their performance using the phantom data. Third, we performed downsampling from high to low grid sized images using linear interpolation and sliding window summation methods and compared their performance against the phantom data. Through this study, we seek to demonstrate inaccuracies that result from naively applying traditional image resizing methods in nuclear scintigraphy and to establish a robust standard for scintigraphic image resizing for future research and developments.

## Method

### Phantom

**Figure 2:**
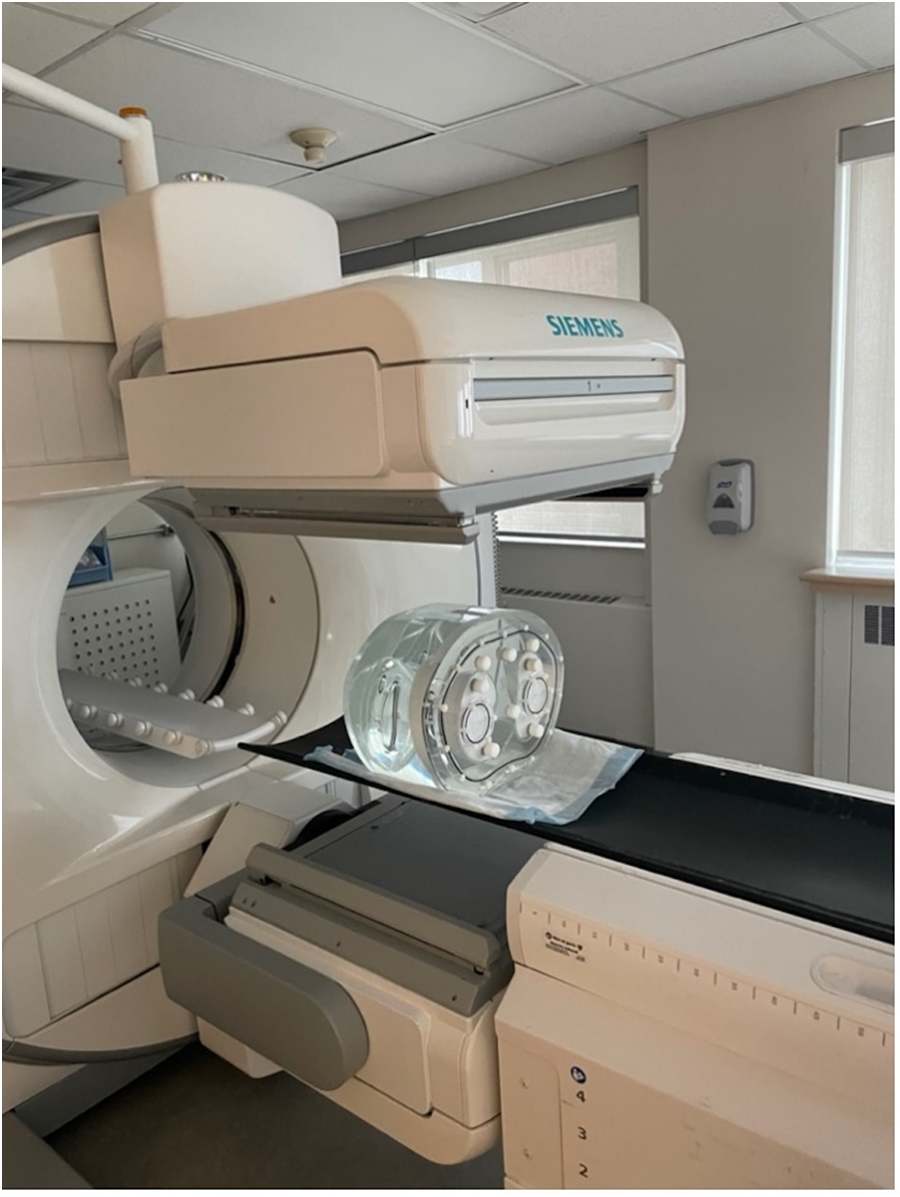
Imaging of the Data Spectrum Anthropomorphic Torso Phantom inside the Siemens Intevo Bold SPECT/CT. The phantom, tailored with dimensions of 45 cm × 33 cm, represents larger patient sizes and anatomical structures pertinent to nuclear lung scans.

This study uses real planar scintigraphic images acquired using a physical phantom (Figure 2). The acquisition protocol was designed to emulate a pulmonary ventilation-perfusion (V/Q) scintigraphy exam using a specially designed Data Spectrum Anthropomorphic Torso Phantom with custom dimensions 45 × 33 cm (left–right × anterior–posterior) that simulates anatomical structures and physiological parameters relevant to nuclear lung scans (14). The phantom included partial (superiorly truncated) lung cavities which were filled with Styrofoam beads to emulate the low density of air-filled lung tissue. An amount of 779 MBq of Technetium-99m (^99m^Tc)-pertechnetate was diluted into approximately 500 ml of tap water, which was then used to fill the space between the Styrofoam beads in the lung cavities. All remaining phantom cavities (thorax and liver) were filled with tap water to emulate soft-tissue attenuation but no activity.

### Image acquisition

All acquisitions were performed in quick succession (within 1 h) to minimize radioactive decay between image sets. Phantom images were acquired with a zoom factor of 1.45 at different spatial grids of 256 × 256, 128 × 128, and 64 × 64 with pixel spacings of 1.64, 3.29, and 6.59 mm^2^, respectively. To easily generate planar images at various count levels, we performed dynamic acquisitions comprised of 100 frames of 1 s duration each, resulting in approximately 11 kcnts/image for a total of 1.1 Mcnts over the whole dynamic acquisition. Dead time was <3% to minimize the impact of count pile-up. Each of the aforementioned acquisitions was performed for the typical six views of a V/Q scan: anterior (ANT), posterior (POST), left anterior oblique (LAO), right posterior oblique (RPO), left posterior oblique (LPO), and right anterior oblique (RAO). Data for the study were acquired with a dual head Siemens Intevo Bold SPECT/CT using low-energy high-resolution collimators (Figure 2). The energy window was set to 140 keV ± 7.5%, corresponding to the photon peak energy of ^99m^Tc.

### Image similarity metrics

In the evaluation phase of our study, we assessed the fidelity of resized images compared to their original counterparts acquired at the target spatial grid size. To achieve this, two metrics were used: the structural similarity index measure (SSIM) and the logarithm of the mean squared error (Log MSE) (15, 16). These metrics serve as the cornerstone of our analysis, allowing us to quantify the extent to which our resizing methods preserve the intrinsic properties of the scintigraphic images.

SSIM offers a comprehensive measure that captures the visual quality of the resized images by evaluating changes in luminance, contrast, and structure. Given the intricate nature of nuclear medicine images, where subtle variations can significantly impact diagnostic outcomes, SSIM's ability to reflect the human visual system's sensitivity to these parameters makes it an invaluable tool in our analysis. The equation for SSIM is as follows:

(2) $$\mathrm{SSIM}(x,y)=\frac{\left(2\mu_{X}\mu_{Y}+c_{1})(2\sigma_{XY}+c_{2}\right)}{\left(\mu_{X}^{2}+\mu_{Y}^{2}+c_{1})(\sigma_{X}^{2}+\sigma_{Y}^{2}+c_{2}\right)}$$

where *X* and *Y* are the two images being compared; $\mu_{X}$ and $\mu_{Y}$ are the average of *X* and *Y*, respectively; $\sigma_{X}^{2}$ and $\sigma_{Y}^{2}$ are the variances of *X* and *Y*, respectively; $\sigma_{XY}$ is the covariances of *X* and *Y*; and $c_{1}$ and $c_{2}$ are scalars to prevent division by zero. These are typically scaled to the dynamic range of the pixel values, L, such that *c*_1_ = 0.01 L and *c*_2_ = 0.03 L.

Complementing the SSIM, the Log MSE metric quantifies the pixel-wise discrepancies between the resized and original images. By calculating the mean of the squared differences between corresponding pixel values and then applying a logarithmic transformation, Log MSE offers a nuanced view of the error distribution. This transformation is particularly adept at highlighting both the high-error and low-error regions within the image, providing a more balanced and interpretable assessment of the resizing method's accuracy. In the context of nuclear scintigraphy, where accurate photon count and noise representation are crucial, the Log MSE metric allows us to critically evaluate whether the resized images deviate from the expected count distribution and noise patterns of the original images.

(3) $$\mathrm{MSE}=\frac{1}{\mathrm{MN}}\overset{M}{\underset{i=1}{\sum }}\;\overset{N}{\underset{\;j=1}{\sum }}\;(X(i,j)-Y(i,j){)}^{2}$$

(4) $$\mathrm{Log}\;\mathrm{MSE}=\;\mathrm{lo}g_{10}(\mathrm{MSE})$$

where

*X* and *Y* are the two images being compared, with X(i,j) and Y(i,j) denoting the pixel values at position (i,j) in the respective images; and *M* and *N* are the dimensions of the images.

Both SSIM and Log MSE were computed after normalizing the images to the range of 0–1 by dividing by their maximum intensity pixel, a step that ensures our evaluation focuses on the relative changes in image characteristics rather than absolute count values. This normalization is especially crucial in our study, as it allows for consistent comparison across images acquired at different grid sizes and count levels.

### Establishing reference similarity curves as a function of count level

**Figure 3:**
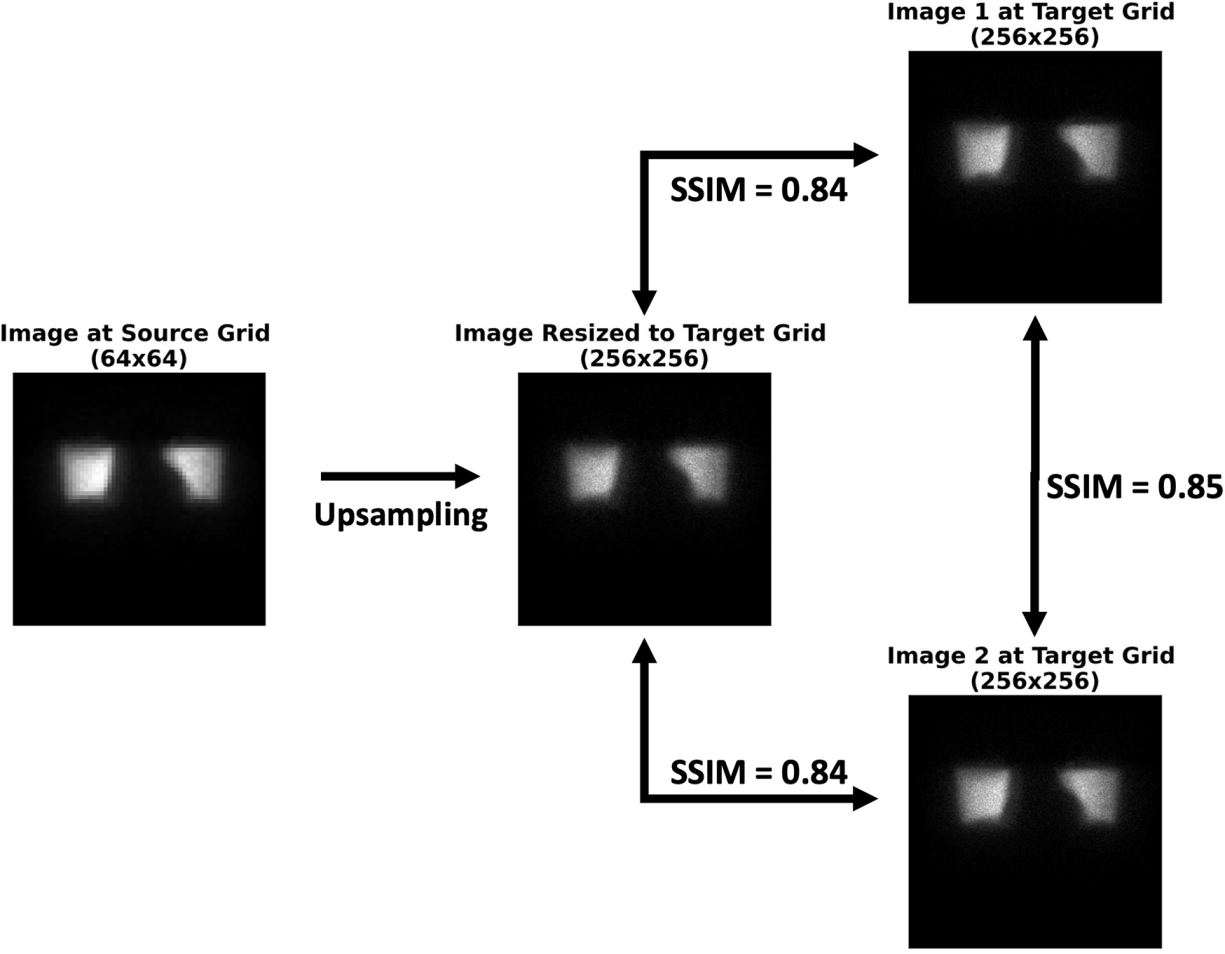
Postulation behind the successful resizing of scintigraphic images. The similarity between the resized image and either image 1 or image 2 at the target grid size is approximately the same as the similarity between images 1 and 2. If this is the case, then the image has successfully been resized. SSIM, structural similarity index measure.

To evaluate and compare resizing strategies, we postulated the following: (1) a successfully resized image of an object should exhibit the same content and noise characteristics as if the image had been acquired on the target grid size, and (2) two images of a given object acquired with the same imaging protocol (i.e., at the same spatial sampling grid) will both have some intrinsic noise and therefore be similar up to a certain point; in other words, the images are not identical, having different permutations of random noise. Therefore, it follows that the similarity between a resized image of an object and another image of the same object at the target grid size should be similar to that of two independently acquired images of the object at the target grid size (Figure 3).

**Figure 4:**
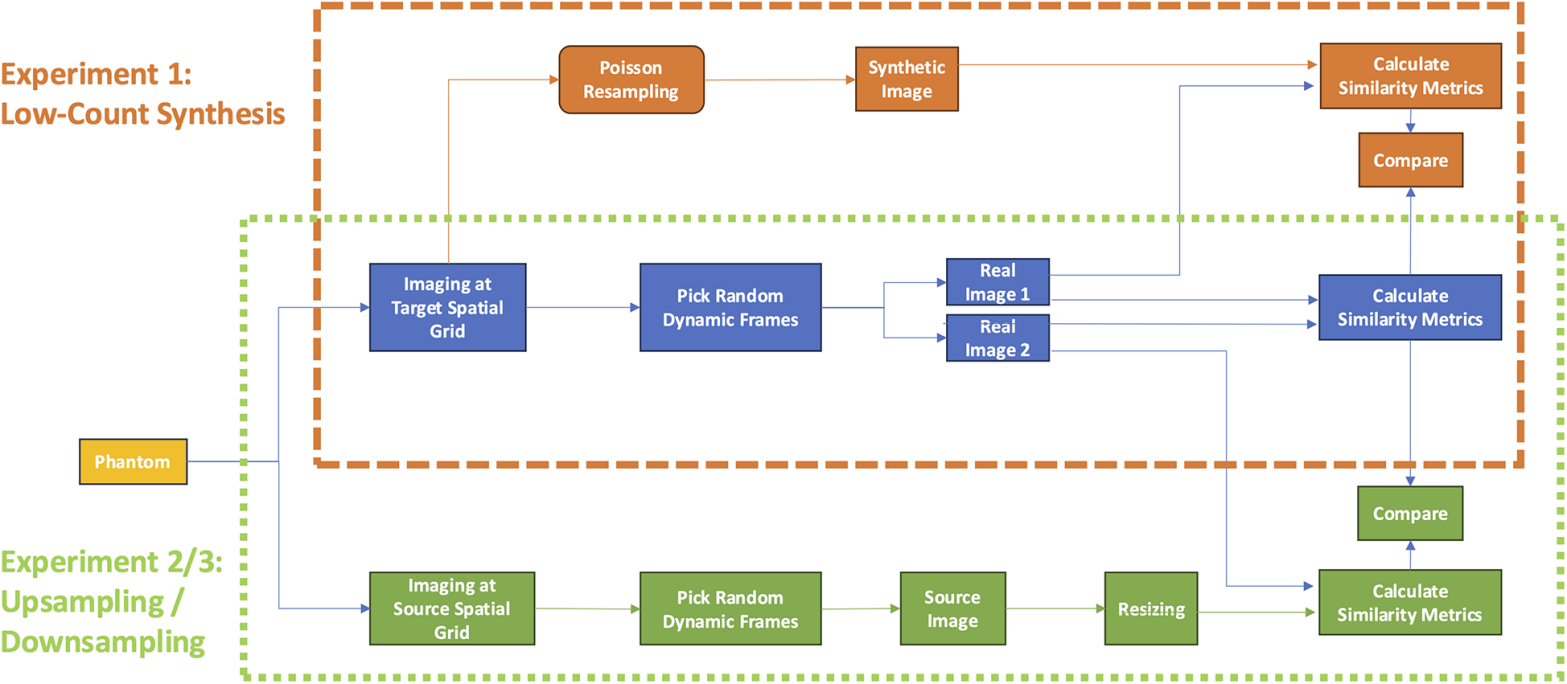
Study design. The first experiment (orange dashed box) evaluated the accuracy of synthetic low-count, Poisson resampled from full-count images (orange workflow) with respect to low-count imaging on the same spatial grid. The second and third experiments (green dotted box) compared the accuracy of upsampling or downsampling images (green) with images acquired at the same count level on the target spatial grid (green workflow). For both experiments, accuracy was evaluated in terms of image-pair similarity metrics SSIM and Log MSE compared to those measured by repeat imaging of the subject at the same spatial grid and count level (blue workflow). SSIM, structural similarity index measure; MSE, mean squared error.

It is worth noting that no image acquired at the target spatial grid size may serve as an absolute reference truth, as each image has some degree of inherent noise. Hence, our analysis rests upon measures of similarity between pairs of images throughout this work. The overall study design is illustrated in Figure 4.

To this end, we sought to derive curves establishing reference measures of similarity achievable between two independent, natively acquired images of the phantom for various count levels and grid sizes (Figure 4, blue). Against these reference curves, we could compare the similarity curves of synthetic low-count, Poisson resampled images (Figure 4, orange) against those of images acquired at the target count level (experiment 1). Likewise, we could then also compare similarity curves between resized images (Figure 4, green) and those acquired at the target spatial grid size (experiments 2 and 3 for upsampling and downsampling, respectively). Consequently, the best resizing method would yield curves that most closely overlap the reference curves.

**Figure 5:**
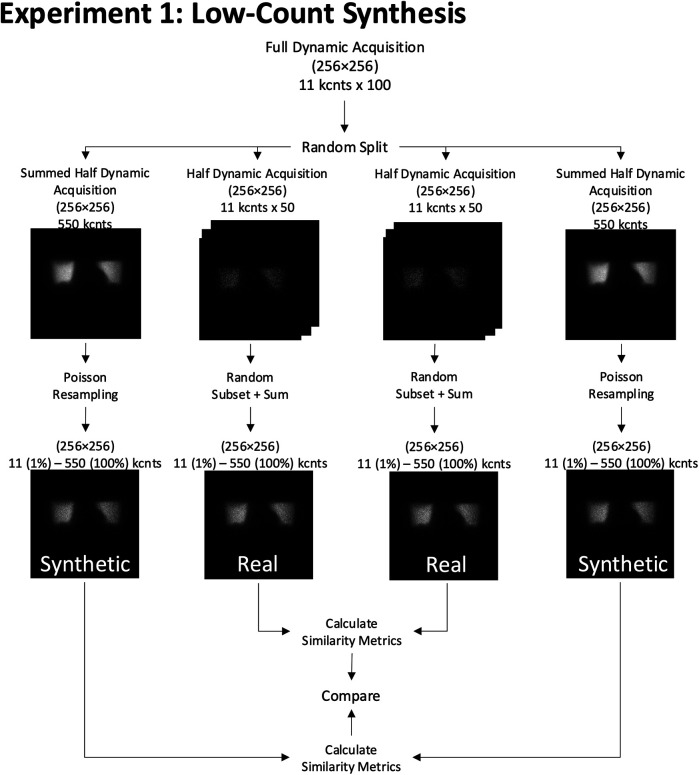
Experiment 1: Low-count synthesis. A simulated full dynamic acquisition experiment conducted on a 256 × 256 grid size to create real and synthetic low-count images.

Time frames from the dynamic image series, containing approximately 11 kcnts each, were randomly split and summed to generate statistically independent images with total counts ranging from 11 to 550 kcnts (Figure 5). To achieve this, combinations of frames were randomly selected without repetition, with the number of frames summed varying depending on the desired total count level. For instance, summing 2 frames resulted in approximately 22 kcnts, while summing all 50 frames yielded the full-count image of 550 kcnts. This approach ensured that each count level was represented by statistically independent images.

Both similarity metrics (SSIM and Log MSE) were calculated for (1) two images acquired at the same grid size and (2) a resized image at a target spatial grid compared to another natively acquired image at the same grid. To generate robust averages and confidence intervals, we repeated this process with 1,000 independent permutations of randomly selected frame combinations for each count level. Reference similarity curves were then generated as a function of the count level between two images acquired at the same grid size.

### Low-count experiment

We also derived a second reference curve by first generating pairs of independent scintigraphic images with the highest possible count value given our dynamic acquisition (here, 550 kcnts) and then synthesizing low-count versions of them with Poisson resampling (Figure 5). While it has already been demonstrated that Poisson resampling effectively yields low-count versions of high-count images that preserve the natural noise characteristics of single-photon emission scintigraphy (9), we decided to verify that two low-count Poisson resampled images would also be as similar to each other as two native low-count planar images. By demonstrating this, we can provide confidence that Poisson resampling is indeed a reliable technique to simulate low-count images when dynamic acquisitions are not available—which is the case in most clinical settings—and that they can be combined with appropriately resized images from other spatial sampling grids. Briefly, a Poisson resampling correction comprises resampling all pixels of the image using a binomial distribution where the initial pixel value constitutes the number of trials and the probability of success in our case is given by the rescaling factor. The method is detailed in the study by White and Lawson (9).

### Upsampling experiment

**Figure 6:**
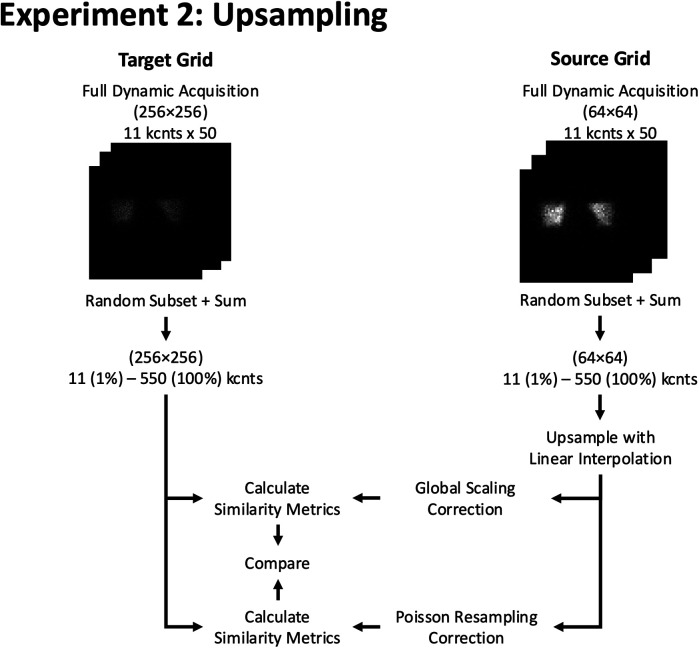
Experiment 2: Upsampling. Example of resizing workflow from a 64 × 64 to a 256 × 256 grid size using 2D phantom planar dynamic acquisition. The similarity metrics of both methods are compared against a pre-determined reference similarity curve at the target (256 × 256) grid size.

Next, we compared the similarity of upsampled images with and without Poisson resampling corrections with the images of matched count level natively acquired on the target grid size (Figure 6). First, we generated an image at a given count level at the lower grid size by randomly selecting and summing the appropriate number of frames from the dynamic sequence at the corresponding sampling grid. Second, we resized the image to the target sampling grid using linear interpolation to attain the higher target grid size. Then, we applied a global scaling correction by a factor corresponding to the increased pixel density [e.g., for 64 × 64 to 256 × 256 upsampling, the rescaling factor was (64/256)^2^ = 1/16] so as to preserve the total number of counts in both images. Third, we applied a Poisson resampling correction per pixel on the resized image to simulate the counting statistical noise that would have been present at the per pixel target count level. A Poisson resampling correction comprises resampling all pixels of the image using a binomial distribution where the initial pixel value constitutes the number of trials and the probability of success in our case is given by the rescaling factor. Fourth, for each upsampled image, we computed the image similarity metrics (SSIM and Log MSE) by comparing it to a corresponding (i.e., same grid size and counts) natively acquired image at the target grid size. To ensure statistical robustness, this process was repeated 1,000 times, using independent permutations of dynamic acquisition images at the lower grid size, to generate confidence intervals for the image similarity curves as a function of count level.

### Downsampling experiment

**Figure 7:**
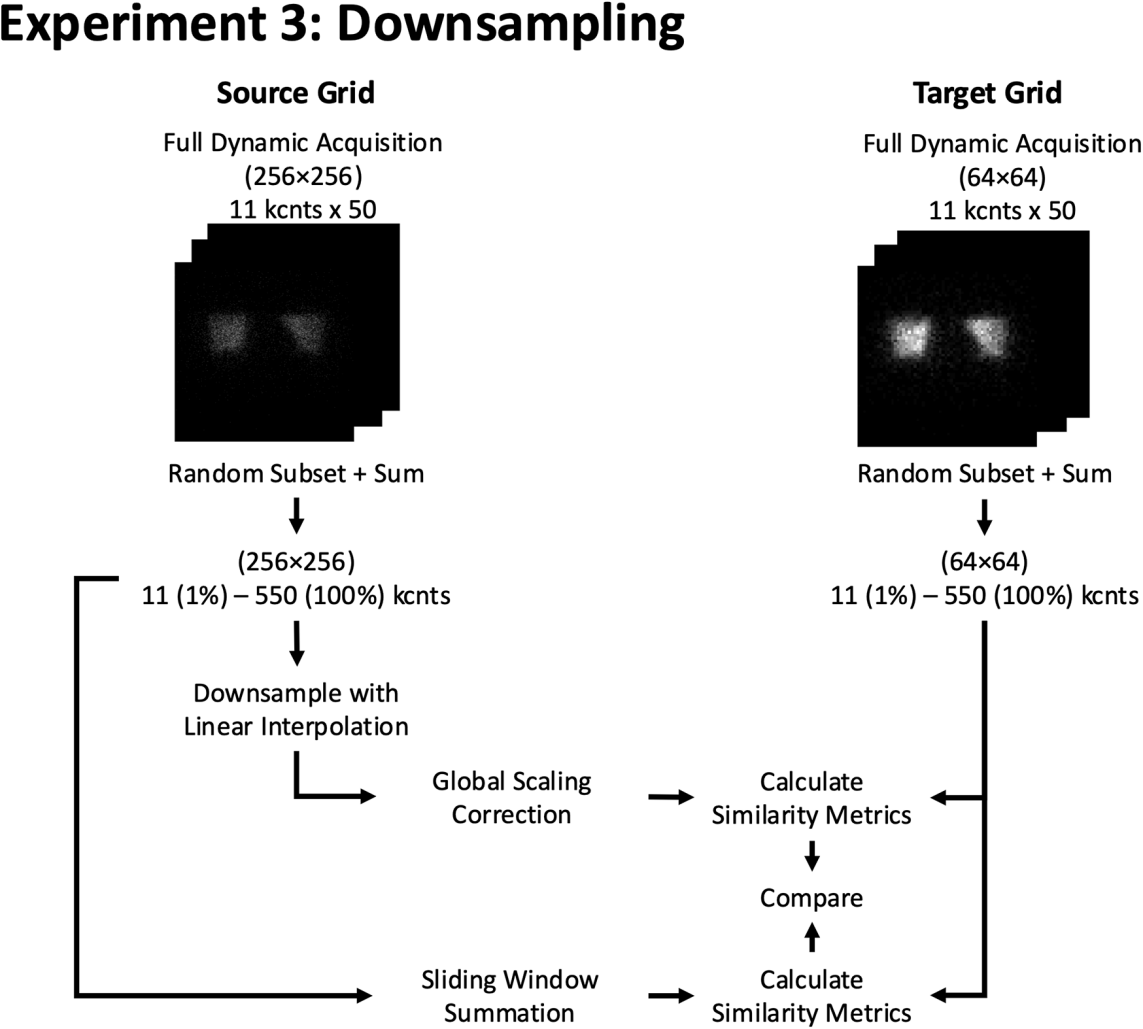
Experiment 3: Downsampling. Example of resizing workflow from a 256 × 256 to a 64 × 64 grid size using 2D phantom planar dynamic acquisition. The similarity metrics of both methods are compared against a pre-determined reference similarity curve at the target (64 × 64) grid size.

Like the upsampling experiment, we compared the similarity of images that were downsampled using either linear interpolation or a sliding window summation method with images of the matched count level natively acquired on the target grid size (Figure 7). We started by producing an image of a given count level by randomly selecting and summing the appropriate number of frames of the dynamic acquisition. The first, naïve, method was to downsample the images by the appropriate factor with linear interpolation to the lower spatial grid.

The second method was a sliding window summation, where the size of the window corresponded to the downsampling factor. Specifically, for a 2× downsampling (e.g., 256 × 256 to 128 × 128), a 2 × 2 non-overlapping sliding window was used to sum the counts from the higher grid size into the lower grid size. Similarly, for a 4× downsampling (e.g., 256 × 256 to 64 × 64), a 4 × 4 non-overlapping sliding window was applied. This approach ensures that the sampling grid of the target image is exactly aligned with the sampling grid of the source image, maintaining consistency with the desired downsampling level and preserving the total counts in the region sampled by the target pixel.

The image similarity metrics were calculated between each downsampled image and another randomly reconstructed native image at the target grid size and counts. To ensure statistical robustness, this process was repeated 1,000 times, using independent permutations of dynamic acquisition images at the higher grid size, to generate confidence intervals for the image similarity curves as a function of count level.

## Results

### Low-count experiment

**Figure 8:**
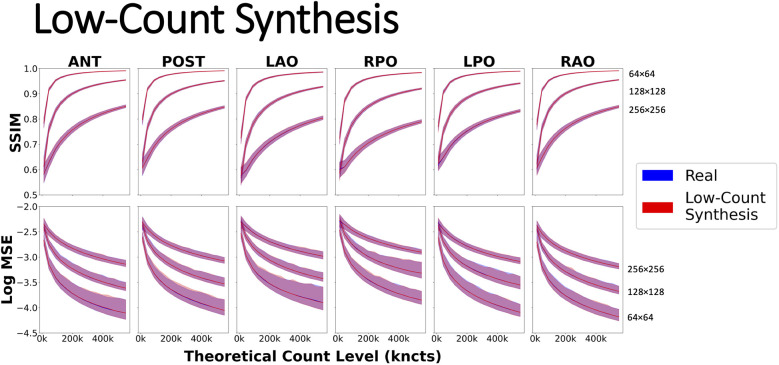
Evaluating similarity metrics SSIM and Log MSE using a dynamic phantom acquisition on six common 2D planar projections: ANT, anterior; LAO, left anterior oblique; LPO, left posterior oblique; POST, posterior; RAO, right anterior oblique; and RPO, right posterior oblique on spatial grids of 64, 128, and 256. Real curves were generated by summing pairs of scintigraphic images up to the highest possible count value of 550 kcnts. Synthetic low-count curves were created by synthesizing low-count versions of 550 kcnts using Poisson resampling correction.

The agreement between similarity curves for real (blue) and synthetic low-count (red) images is evident in Figure 8 with nearly perfect overlap across all projections, count levels, and grid sizes, confirming that the synthesized (count reduced) images accurately modeled the statistical noise associated with low-count images. Another general finding was that image similarity increased (i.e., higher SSIM and lower MSE) as the spatial sampling grid decreased.

### Visual inspection of upsampled and downsampled images

**Figure 9:**
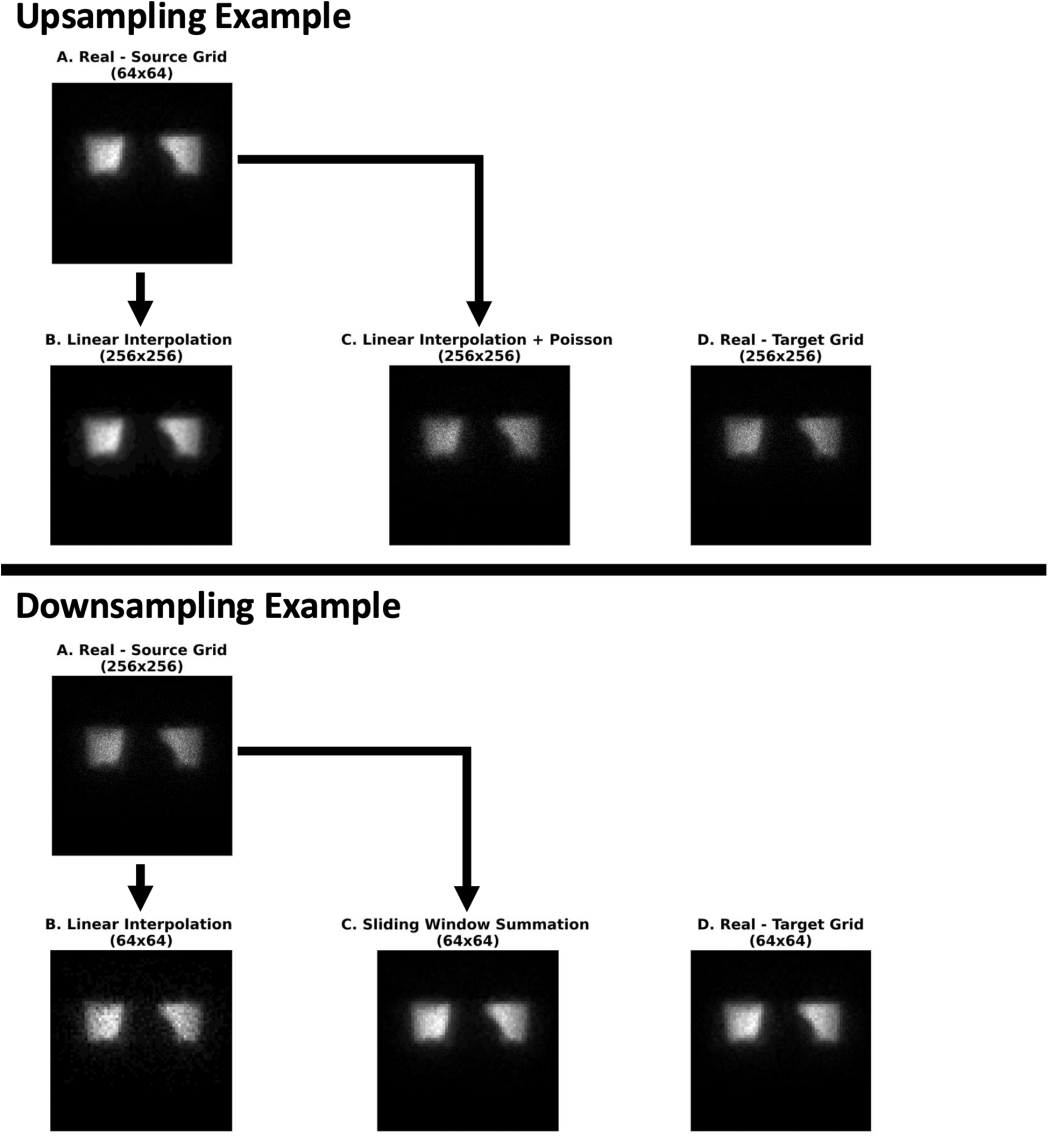
An illustrative example of the effect of the various upsampling (Experiment 2) and downsampling (Experiment 3) methods on image similarity with respect to real scintigraphic images at approximately 105 kcnts. (Experiment 2) From left to right: (A) a real (64 × 64) image at the source grid upsampled with only linear interpolation (B) followed by a Poisson resampling correction (C). These were compared with a real (256 × 256) image at the target grid generated from the raw dynamic acquisition (D). (Experiment 3) From left to right: (A) a real (256 × 256) image at the source grid downsampled with linear interpolation (B) and a sliding window summation (4 × 4 window) (C). These were compared against a real (64 × 64) image at the target grid (D) image reconstructed from the dynamic acquisition. All images were rendered in grayscale with their intensity levels scaled according to their respective minimum and maximum pixel values.

The top part of Figure 9 (Upsampling) demonstrates the upsampling of an image from 64 × 64 to 256 × 256 and the effect of resizing on image similarity. As can be seen, upsampling with naïve linear interpolation maintains the original (B) contrast of high- to low-count areas of the lower grid sized image (A), which is less pronounced on the higher grid sized image (D). The application of a Poisson resampling correction (C) visually seems to restore the natural image contrast characteristic Poisson noise seen in the real image at the target higher grid size (D).

In the bottom part of Figure 9 (Downsampling), the source image (A) is downsampled from 256 × 256 to 64 × 64 using two methods: linear interpolation (B) and a sliding window summation with a 4 × 4 window (C). Both methods appear to preserve the contrast between high- and low-count areas. However, the sliding window summation method produces noise characteristics that more closely resemble the images natively acquired at the target lower grid (64 × 64) (D).

### Effect of resizing methods on image similarity

**Figure 10:**
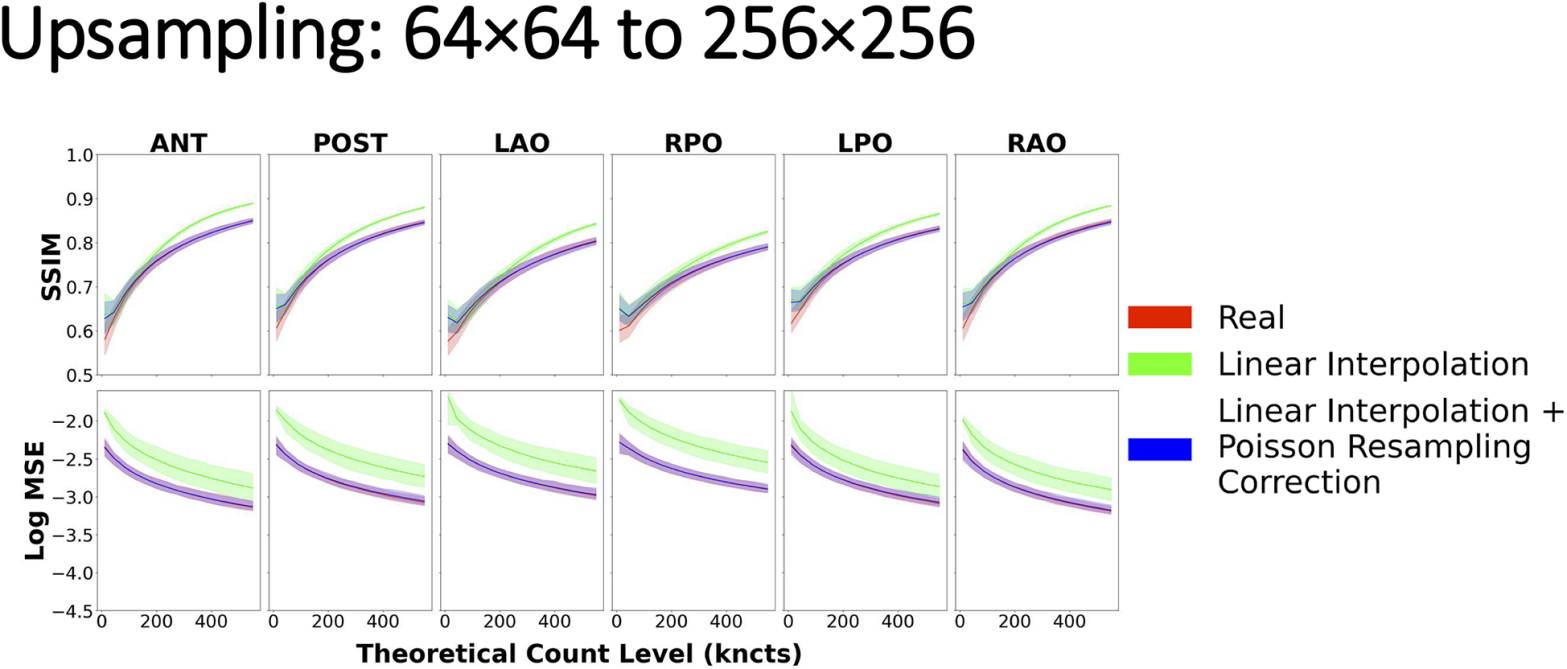
Image similarity metrics for 64 × 64 images upsampled to 256 × 256 with a reference curve from the real phantom experiment data. Linear interpolation + Poisson resampling correction overlays the real reference almost perfectly across all count levels, indicating good agreement, while the linear interpolation alone does not.

Upsampling with naïve linear interpolation yielded image similarity curves that deviated substantially from the real reference curve regardless of the source or target spatial grid, count level, and projection, as shown in Figure 10. In particular, when compared with scintigraphic images natively acquired on the target spatial grid, upsampled images with naïve linear interpolation produced higher SSIM and higher MSE than the real reference curves from the phantom data. The deviations were more marked (i.e., less overlap of the confidence intervals) when upsampling from either 64 × 64 or 128 × 128 to the highest grid of 256 × 256, whereas there was more overlap of the similarity curves when upsampling from 64 × 64 to 128 × 128 (Figure 10, Supplementary Figures S1, S2). However, following the Poisson resampling correction, the similarity curves realigned with the reference curve with respect to the means and confidence intervals for each target spatial grid, count level, and projection.

**Figure 11:**
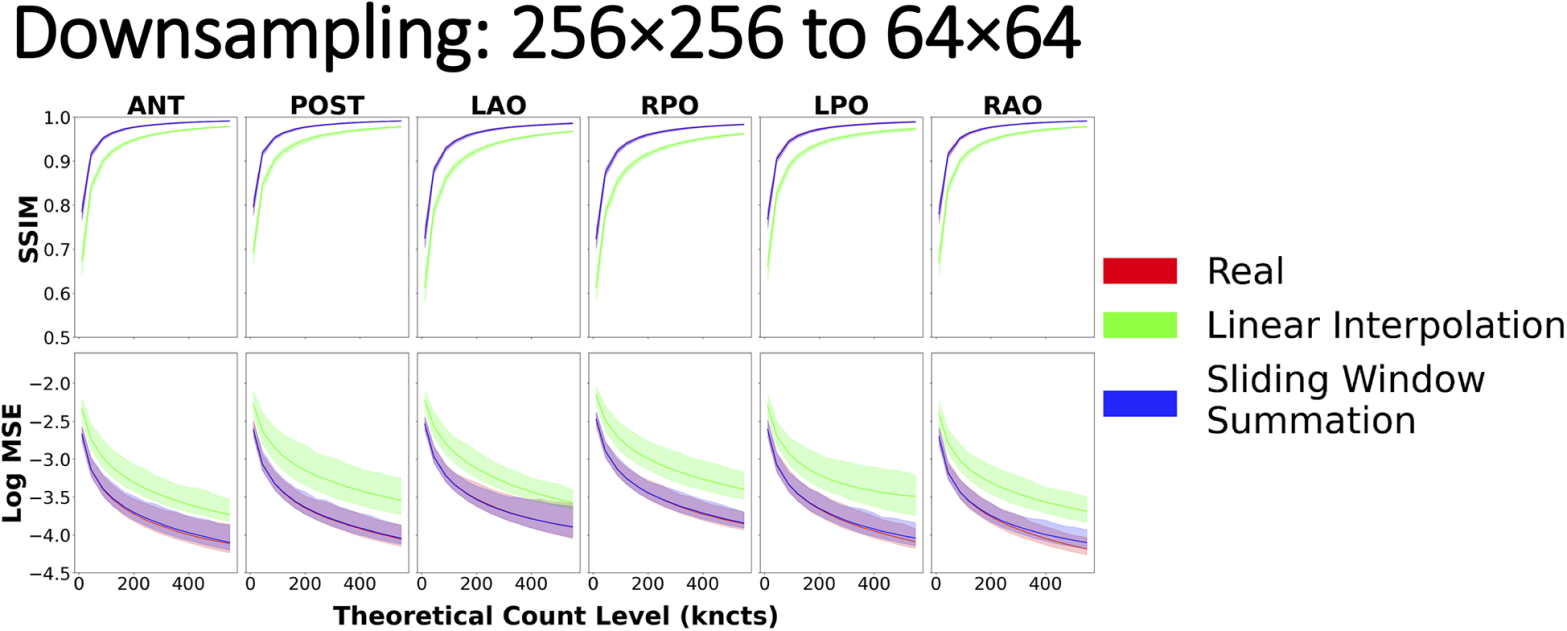
Image similarity metrics for 256 × 256 images downsampled to 64 × 64 with a reference curve from the real phantom experiment data. Real phantom data and sliding window summation overlay almost perfectly across all count levels, indicating good agreement, while linear interpolation alone does not.

With regards to downsampling, the most striking result was that when resizing by a factor of 2 (i.e., from 256 × 256 to 128 × 128 or from 128 × 128 to 64 × 64), linear interpolation and sliding window summation methods yielded similar similarity curves, both of which overlaid nearly perfectly on the reference curve (Supplementary Figures S3, S4). However, when downsampling from 256 × 256 to 64 × 64, naïve linear interpolation yielded similarity curves that significantly deviated from the reference curve (Figure 11). In this case, when compared with scintigraphic images natively acquired on the target spatial grid (64 × 64), downsampled images with naïve linear interpolation resulted in lower values for SSIM and higher MSE with respect to the reference curve. Sliding window summation, however, produced a high level of agreement with the real data reference curve.

## Discussion

In this study, we conducted three distinct experiments with the goal of preserving the noise and statistical properties inherent to scintigraphic images (9). First, we used the phantom data to establish reference similarity curves for different projections, image grid sizes, and image count levels. These curves were critical for validating the statistical properties of subsequent low-count, upsampling, and downsampling experiments. The first experiment, the low-count experiment, generated synthetic low-count images from high-count dynamics and validated their similarity curves against the reference curves from the phantom. Our findings from the low-count experiment corroborate the results by White and Lawson (9), affirming that count statistics in scintigraphic images are best modeled and preserved using Poisson-based statistics (6).

The second experiment, upsampling, demonstrated that naïve linear interpolation distorts noise characteristics and fails to accurately replicate the statistical properties of high grid sized images. The application of Poisson resampling correction effectively addressed these limitations, preserving both counts and associated noise. Finally, the third experiment, downsampling, demonstrated that sliding window summation consistently preserved total counts and noise properties, outperforming linear interpolation, especially at larger downsampling factors.

By examining upsampling and downsampling techniques used for resizing scintigraphic images and their impact on preserving image noise properties, our goal was to guide the research community on proper methodology. While potentially applicable to many image processing applications, we believe that these lessons are particularly applicable to nuclear medical imaging where image pre-processing frequently includes image resizing.

### Upsampling

In the case of image upsampling, we demonstrated that the naïve application of linear interpolation violates inherent noise characteristics of scintigraphic images. Specifically, as images undergo greater degrees of upsampling [i.e., from a 64 × 64 to a 256 × 256 (Figure 10) grid as opposed to 128 × 128 to 256 × 256 (Supplementary Figure S1)], the difference in similarity becomes more evident. These results can be partly explained by the mechanism by which linear interpolation operates (3). In the first order, linear interpolation by a factor of 2 averages pixel intensities in a small vicinity, close to a neighborhood of 2 × 2 pixels. However, as the scaling factor increases (64 × 64 to 256 × 256) (Figure 10), linear interpolation no longer includes in its average those pixels that are further than in the immediate vicinity of the center of the interpolated pixel.

Furthermore, it is evident that solely relying on interpolation does not provide an accurate representation of the target grid sized image, especially in relation to its noise characteristics. Hence, the recommended method for image upsampling is as follows: first, resize the image with linear interpolation to the new sampling grid; second, apply a Poisson resampling correction by resampling the linearly interpolated image, rounded to the nearest integer, with a binomial distribution where the interpolated integer pixel value constitutes the number of trials and the probability of success. The probability in our case is given by the ratio between the old and new pixel spacing (9).

Perhaps surprisingly, interpolation-based upsampling resulted in higher SSIM values (green) than those achieved when comparing two images acquired at the target grid size (red), as shown in Figure 10 and Supplementary Figures S1, S2. This may be explained by the interpolation being performed from larger pixels, with more counts and less relative noise being reused to derive many small pixels in the upsampled image. Consequently, the local variance component of the SSIM equation in the upsampled image is reduced (as confirmed in Figure 9, Upsampling, image B) leading to inflated SSIM measures. Perhaps, counterintuitively, in this scenario, a higher SSIM does not correspond to a more realistic upsampled image, simply because the SSIM deviates from the SSIM measured between two images acquired at the target grid size, which goes against our postulation of a successful resizing.

### Downsampling

For downsampling images, we explored two techniques: linear interpolation (3) and sliding window summation (17). Both methods yielded similar similarity metrics (and in agreement with reference values) when downsampling by a factor of 2 [i.e., from 256 × 256 to 128 × 128 (Supplementary Figure S3) or from 128 × 128 to 64 × 64 (Supplementary Figure S4)]. However, when downsampling images by a larger factor [i.e., from 256 × 256 to 64 × 64 (Figure 11)], linear interpolation deviated from the real reference curve. The observed outcomes can be attributed, in part, to the principles governing linear interpolation when downsampling by a factor of 2. In this scenario, the position of the pixels in the downsampled image will fall perfectly in the center of a 2 × 2 window in the original image, resulting in the average of all four values in the window. By introducing a global scale correction to the linearly interpolated downsampled image, we recover the result of using a 2 × 2 sliding window summation. However, with an increasing scaling factor, linear interpolation begins to exclude pixels that are not in the immediate proximity of the center of the interpolated pixel. In contrast, sliding window summation accounts for all pixels in the window for all sizes. Furthermore, since summing the original image pixel values mimics how a gamma camera would have aggregated photon counts within a larger pixel size, the resulting image necessarily has preserved total image counts and the correct noise characteristics. Hence, the recommended procedure for downsampling nuclear scintigraphic images is to apply sliding window summation rather than resampling with interpolation.

### Implications for nuclear medicine image processing

One domain where resizing nuclear medicine images can have a profound impact is AI development. Indeed, there has been a strong emphasis on leveraging precise and representative data for AI model training and evaluation. The pitfalls of relying on inadequately simulated or non-representative data have been underscored in current literature (18). When machine learning models are trained on datasets that do not encompass the complexities of real-world scenarios, there is an inherent risk of these models yielding untrustworthy or inaccurate results (19, 20). This disjunction between training data and real-world samples can severely impede a model's proficiency in image interpretation, directly influencing clinical decisions and patient outcomes. Moreover, models fed with non-representative data often demonstrate excellent performance during validation phases using similar datasets, showcasing high accuracy and precision. However, their efficiency might be compromised in real-world applications due to overfitting, making them less versatile and responsive to diverse clinical data (21).

Image resizing can also play an important role in multimodal co-registration where a source image is often resampled to the target image's reference frame and thus pixel/voxel size. Therefore, in cases where planar scintigraphy images are the source image, care should be taken to adjust the counts in the resulting image to account for the change in pixel/voxel spacing. This could be relevant, for instance, in registering scintigraphy bone scan data onto x-ray images for anatomical localization of metabolic abnormalities to harmonize inter-patient observations (22). This is especially crucial if photon count statistics (mean, max, standard deviation, etc.) were to be manually extracted from these registered images and used as features in a downstream machine learning or radiomics task (23, 24).

### Limitations

Despite our unequivocal results on the proper methods for resizing nuclear medicine images, our study is not without its limitations. First, we investigated convenient resizing factors of 2 and 4 on the most commonly used sampling grids in nuclear medicine scintigraphic applications (i.e., 256 × 256, 128 × 128, and 64 × 64). While upsampling by non-integer factors can still be accomplished with the recommended Poisson resampling correction, the correct procedure for downsampling by non-integer factors was not demonstrated in our study. Nevertheless, this work demonstrates the importance, and general concepts, to be applied for the more general case. Namely, downsampling requires moving summation windows that represent the image area covered by each target pixel. In cases where the source and target grids do not perfectly align, spatially varying weighted averages may be applied. Alternatively, upsampling followed by downsampling stages may be combined.

Our study utilized phantom images to simulate a lung perfusion scan; however, because our methodology was grounded in the fundamental principles of Poisson counting statistics and scintigraphy image acquisition, this should carry over to any real-world clinical applications in nuclear medicine scintigraphy. While we acknowledge that other interpolation techniques exist, for the scope of this study, we focused primarily on linear interpolation due to its prevalent use in the field. Another potential criticism is that we did not directly assess the potential effects of resizing on 3D single-photon emission computed tomography (SPECT) reconstructions, as there are many post-processing steps in reconstructing tomographic volumes from sinograms (such as spatial smoothing), that may invalidate our assumptions and methodology. In the case of positron emission tomography (PET) imaging, since voxel units are typically Bq/ml, which is a measure of activity density, they do not directly encode event count, further complicating the estimation of voxel count statistics. Furthermore, we did not investigate how to adapt our recommendations to non-linear registrations where local parts of the image have shrunk or expanded independently. While in theory, one could locally apply Poisson resampling corrections in enlarged areas or sum counts in contracted areas, this technical development was outside the scope of this study yet merits further attention.

Both SSIM and Log MSE metrics were calculated after normalizing the images to the range of 0–1, based on their maximum intensity. This normalization ensured consistent comparison across images with varying spatial resolutions, grid sizes, and count levels. While normalization by maximum intensity is commonly used, we acknowledge it may amplify noise in low-count regions, potentially affecting similarity metrics. An alternative approach, such as normalizing by the total counts across the entire image or a large region, could mitigate such issues.

While this work was performed using traditional cameras with scintillation crystals, photomultiplier tubes, and Anger logic, it deals with scintigraphic images of counted photon events. The same lessons may be applied to planar images acquired with semiconductor-based detectors, as these images contain the information. The pixel statistics in both cases are governed by a Poisson distribution. On Anger cameras, the grid size is a variable parameter used to define the pixels into which arbitrarily located detection events within the crystal are binned. In contrast, semiconductor detectors have hardware-defined pixel grids. Image downsampling techniques such as those described in this work (i.e., moving summation windows) may be applied to generate images at grid sizes other than those intrinsic to the hardware.

Finally, the use of a physical phantom may be criticized for lacking physiologic realism. We partially tried to compensate for this using six different projections. Nevertheless, having consistent patterns of activity distribution was fundamental to this research methodology and, therefore, the use of living subjects, in which tracer distribution and motion are to be expected, would not be appropriate. Similar work is possible with numerical phantoms, but the empirical evidence in our methodology provides greater confidence in its practical relevance for clinical systems.

## Conclusion

Image resizing is a common process in medical imaging, however, many neglect to reflect on its finer nuances. We make the case that in the context of nuclear image scintigraphy, one must take care to adopt methods that preserve total image counts and maintain realistic image noise properties. We provide a recipe for simple upsampling and downsampling of scintigraphic images to enable the scientific community to properly perform image rescaling operations in practice.

## Data Availability

The raw data supporting the conclusions of this article will be made available by the authors upon request, without undue reservation.
