# Supplementary material for: The effect of resizing on the natural appearance of scintigraphic images: an image similarity analysis
Source: Front Nucl Med. 2025 Feb 6;4:1505377. doi: 10.3389/fnume.2024.1505377 (PMC11839826; doi:10.3389/fnume.2024.1505377)
Supplement: Supplementary file 1 [file Supplementaryfile1.docx]

# Supplementary Material

The following results of the phantom upsampling and downsampling experiments show the same patterns as in Figures 10 and 11 respectively. The figures correspond to different source and target image sampling matrix sizes. Figures 10 and 11 correspond to a resizing factor of 4, while the figures below correspond to a resizing factor of 2.

When upsampling, Linear Interpolation must be followed by Poisson Resampling Correction to achieve statistical noise emulating a real change in count statistics associated with smaller pixel sizes. This is consistent across both resizing factors.


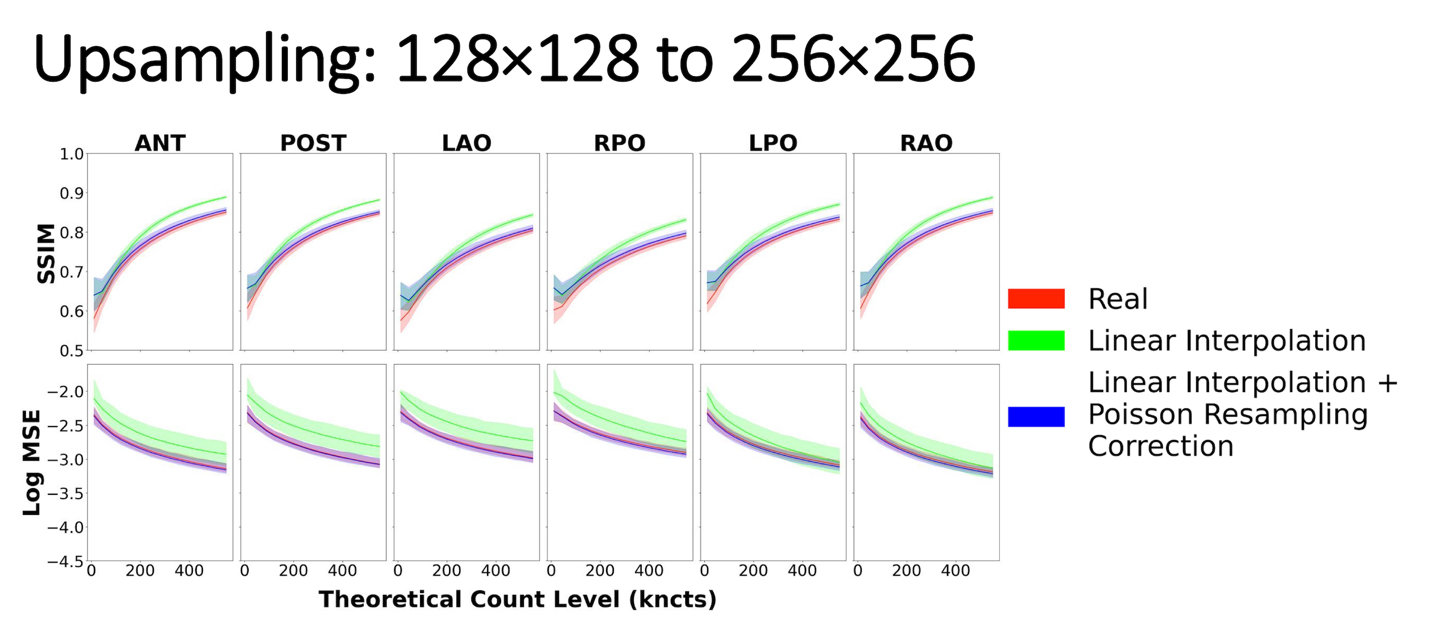
Supplementary Figure 1: Image similarity metrics for 128×128 images upsampled to 256×256 with reference curve from the Real, experimentally measured phantom data. The Linear Interpolation + Poisson Resampling Correction overlay almost perfectly with the reference Real data across all count levels, while the Linear Interpolated images alone do not.


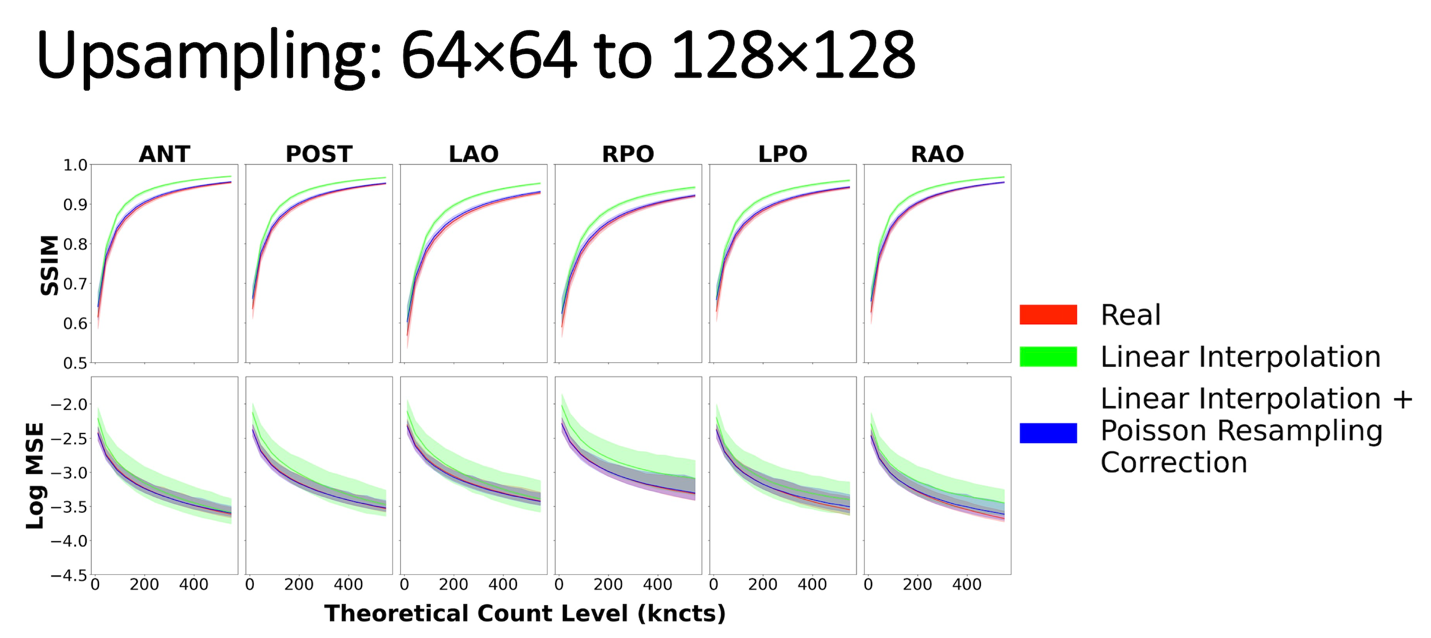


Supplementary Figure 2: Image similarity metrics for 64×64 images upsampled to 128×128 with reference curve from the Real, experimentally measured phantom data. The Linear Interpolation + Poisson Resampling Correction overlay almost perfectly with the reference Real data across all count levels, while the Linear Interpolated images alone do not.

When downsampling, a sliding window summation better emulates the increase in pixel count statistics associated with larger pixel sizes (Figure 11). The exception is when downsampling by a factor of 2 (below two figures) in which case, the Linear Interpolation and Sliding Window Summation are essentially identical operations, producing nearly identical similarity results regardless of resampling method.


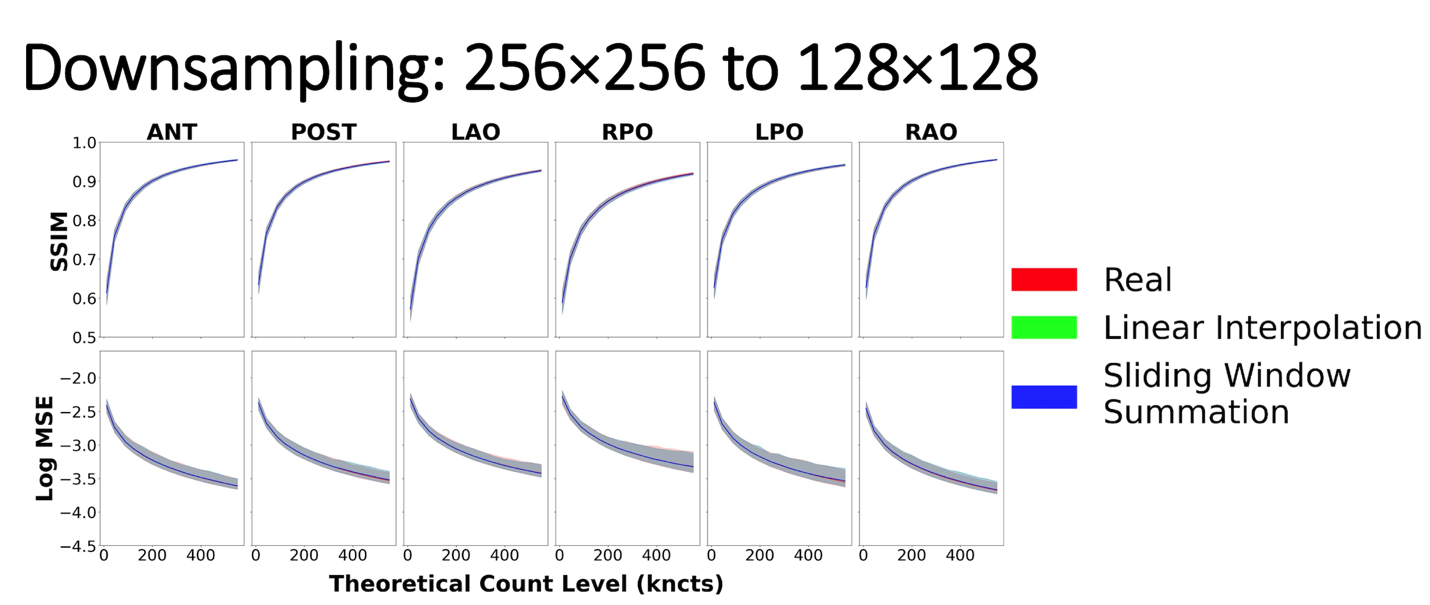
Supplementary Figure 3: Image similarity metrics for 256×256 images downsampled to 128×128 with reference curve from the Real, experimentally measured phantom data. The Sliding Window Summation overlay almost perfectly with the reference Real data across all count levels, as do the Linear Interpolated images.


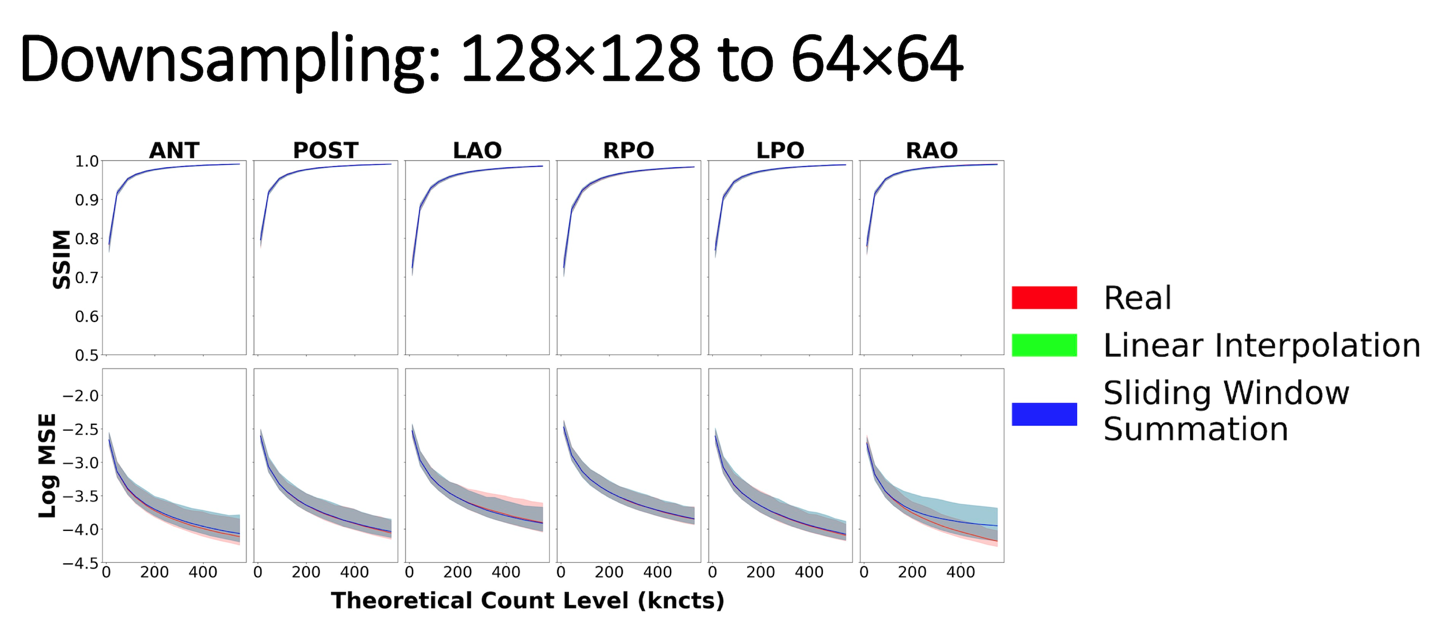
Supplementary Figure 4: Image similarity metrics for 128×128 images downsampled to 64×64 with reference curve from the Real, experimentally measured phantom data. The Sliding Window Summation overlay almost perfectly with the reference Real data across all count levels, while the Linear Interpolated images alone do not.
